# Oncolytic Immunotherapy: Conceptual Evolution, Current Strategies, and Future Perspectives

**DOI:** 10.3389/fimmu.2017.00555

**Published:** 2017-05-15

**Authors:** Zong Sheng Guo, Zuqiang Liu, Stacy Kowalsky, Mathilde Feist, Pawel Kalinski, Binfeng Lu, Walter J. Storkus, David L. Bartlett

**Affiliations:** ^1^University of Pittsburgh Cancer Institute, Pittsburgh, PA, USA; ^2^Department of Surgery, University of Pittsburgh School of Medicine, Pittsburgh, PA, USA; ^3^Department of Surgery, CCM/CVK, Charité – Universitaetsmedizin Berlin, Berlin, Germany; ^4^Department of Immunology, University of Pittsburgh School of Medicine, Pittsburgh, PA, USA; ^5^Department of Dermatology, University of Pittsburgh School of Medicine, Pittsburgh, PA, USA

**Keywords:** immunogenic cell death, ICD inducer, antigen, cross-presentation, immune checkpoint blockade, antitumor immunity, T cells, combination

## Abstract

The concept of oncolytic virus (OV)-mediated cancer therapy has been shifted from an operational virotherapy paradigm to an immunotherapy. OVs often induce immunogenic cell death (ICD) of cancer cells, and they may interact directly with immune cells as well to prime antitumor immunity. We and others have developed a number of strategies to further stimulate antitumor immunity and to productively modulate the tumor microenvironment (TME) for potent and sustained antitumor immune cell activity. First, OVs have been engineered or combined with other ICD inducers to promote more effective T cell cross-priming, and in many cases, the breaking of functional immune tolerance. Second, OVs may be armed to express Th1-stimulatory cytokines/chemokines or costimulators to recruit and sustain the potent antitumor immunity into the TME to focus their therapeutic activity within the sites of disease. Third, combinations of OV with immunomodulatory drugs or antibodies that recondition the TME have proven to be highly promising in early studies. Fourth, combinations of OVs with other immunotherapeutic regimens (such as prime-boost cancer vaccines, CAR T cells; armed with bispecific T-cell engagers) have also yielded promising preliminary findings. Finally, OVs have been combined with immune checkpoint blockade, with robust antitumor efficacy being observed in pilot evaluations. Despite some expected hurdles for the rapid translation of OV-based state-of-the-art protocols, we believe that a cohort of these novel approaches will join the repertoire of standard cancer treatment options in the near future.

## Introduction

Successful cancer therapy using oncolytic viruses (OV) is predicated on at least three major (and coordinate) mechanisms of action. Among them, the first is the direct infection of cancer cells and endothelial cells and the subsequent oncolysis of these cells in the tumor microenvironment (TME). The second involves indirect effects of necrosis/apoptosis of uninfected cancer cells and associated endothelial cells in the tumor-associated vasculature leading to reduced angiogenesis (1–3). Finally, antitumor (and antiviral) immunity is elicited/expanded by the OV as a consequence of improved antigen cross-priming and recruitment of immune cells into the TME. More than 10 years ago, most, if not all, investigators thought that the direct oncolysis was the only major mechanism by which OVs inhibited tumor growth, leading to the terminology of “oncolytic virotherapy,” coined by Kirn in 2001 (4). Later, investigators discovered that the host immune response was critical to the antitumor efficacy of oncolytic virotherapy. Briefly, this has been shown through multiple approaches including the use of (1) an OV encoding a tumor antigen to potently activate therapeutic T cell responses (5); (2) reovirus infection of tumor cells to prime antitumor immunity capable of reducing metastatic disease burden (6); and (3) CD8^+^ T cell depletion resulting in the loss of efficacy associated with OV-based treatment (7). Thus, OV represents a novel form of immunotherapy (8), with Rommelaere and associates formally advocating the term “oncolytic immunotherapy” in their article published in 2011 (9). Since then many other investigators, including our group, have adopted this terminology (10–14). As most investigators have discovered, single modality therapies (including OV) may be insufficient to effect cure in the cancer setting, mandating the development of combination protocols implementing antitumor agents capable of yielding additive or synergistic antitumor benefits. Our discussion will focus on combination regimens likely to yield superior antitumor immunity associated with improved treatment outcomes.

## The Conceptual Shift from Virotherapy to Oncolytic Immunotherapy

Although the use of viruses as oncolytic agents has a rich history, the application of genetically engineered viruses to selectively target cancer cells is a relatively recent adaptation (15). The first research article reporting the use of a genetically engineered OV was published by Martuza and colleagues in 1991 (16), in which the authors showed that infection with a thymidine kinase (*tk*) gene-deleted herpes simplex virus (HSV) led to the death of multiple human glioma cell lines, as well as, primary cultures of human glioma cells. Furthermore, they demonstrated that intratumoral inoculation of the *tk* gene-deleted HSV led to the slowed growth of human glioma xenografts in SCID mice and to the extended overall survival of these animals. In most early studies, it was thought that the major mechanism associated with OV treatment benefit involved selective viral replication in cancer cells and consequent tumor cell lysis or apoptosis (17). For example, an oncolytic HSV-mediated tumor inhibition showed equivalent effects in immune-competent and immune-incompetent mice, suggesting that viral oncolysis and not the host immune response was the primary mechanism linked to tumor destruction (18). Thus, investigators at that time paid significant attention to remove viral genes that would limit tumor cell lysis or apoptosis, such as the adenovirus gene encoding E1B-19 kDa protein (19) or vaccinia virus (VACV) genes for SPI-1 and SPI-2 (20). In addition, to accentuate such pathways, OVs commonly incorporated suicide genes or genes promoting apoptosis such as a suicide gene encoding purine nucleotide phosphorylase (21), apoptosis-inducing gene TRAIL (22), or tumor suppressor gene TP53 (23, 24).

Yet, investigators repeatedly noticed the critical role of antitumor T cells in OV-mediated therapeutic efficacy in their studies. In 1999, Martuza and associates found that infection of established CT26 tumors in mice using an HSV-1 OV G207 led to the generation of highly specific, systemic antitumor immunity (25). Later, Vile and associates demonstrated that tumor infection by oncolytic reovirus primes adaptive antitumor immunity (6). They also showed that CD8^+^ T cells played a critical role in the therapeutic efficacy of intratumorally delivered vesicular stomatitis virus (VSV), with these T cells specific for immunodominant epitopes derived from both viral- and tumor-associated target proteins (5). The authors utilized two approaches to show the important roles of CD8^+^ T cells in this therapy. First, by increasing the circulating levels of tumor antigen-specific T cells using adoptive T cell transfer, in combination with intratumoral virotherapy, the investigators observed significantly enhanced therapeutic efficacy over either monotherapy. Second, the integration of a tumor-associated antigen (TAA) within the oncolytic VSV was found to increase the level of activation of naive T cells recognizing that antigen, in association with enhanced antitumor activity. As a consequence, they termed their approach an “oncolytic immunovirotherapy” (5). Zhang and associates showed that tumor destruction after delivery of an HSV2-based OV (FusOn-H2) *in vivo* induced potent antitumor immune responses in a syngeneic neuroblastoma model (26, 27). Even UV-inactivated Sendai virus (particle) was shown to eradicate tumors by promoting antitumor immunity as a consequence of blocking the immunosuppressive action of regulatory T cells (Tregs), believed to be mediated *via* the viral particle-induced secretion of IL-6 from activated dendritic cell (DC), independent of cancer cell infection (28). In addition, investigators have developed OV armed with genes to stimulate immune responses, as showcased by T-VEC, originally constructed and tested in 2003 (29). On the basis of an increasing body of evidence, we and others concluded that OVs are promising novel immunotherapeutic strategies (8, 30). More recently, Bhat et al. have coined the term “oncolytic immunotherapy” in reference of their study of oncolytic parvovirus to activate NK cells capable of killing cancer cells in 2011 (9). Hemminki and associates have also applied this term in their clinical study using an oncolytic AdV expressing CD40L, where they observed induction of potent tumor antigen (surviving)-specific CD8^+^ T cells associated with robust antitumor activity (10). Many in the field have now adopted this nomenclature as it is believed to best reflect the intrinsic immunologic mechanisms of action associated with this class of novel antitumor agents (8, 10–14, 30, 31).

## Current Strategies in Oncolytic Immunotherapy

In this section, we will introduce the concept of tumor immunogenic cell death (ICD), how OVs induce ICD, and how this may lead to the development of potent, durable antitumor immune responses in treated individuals. We will then discuss current concepts for preclinical studies and the clinical implementation of OVs as monotherapies or combination protocols integrating a range of chemotherapeutic agents or immunomodulatory compounds.

### Immunogenic Cell Death (ICD)

In a previous review, we summarized the developmental concept of ICD and key features of this type of cell death that leads to robust antitumor immune responses (12). Here, we will update this important and evolving paradigm and discuss new findings related to the role of OV-associated ICD with the development of therapeutic antitumor immunity.

Intrinsic to this discussion is the question of how the immune system senses danger associated with pathogenic infection or the development of a pathologic state (such as cancer). As Janeway summarized, the immune system distinguishes self from non-self “events” based on the surveillance of differences and danger signals predicated on so-called immune signals 1, 2, 3, and 0 (32). Signal 0 derives from pathogens and is now called pathogen-associated molecular patterns (PAMPs). In 1994, Matzinger proposed that danger signals are also communicated from the inside of dying cells, i.e., damage-associated molecular patterns (DAMPs) (33, 34). In recent years, ICD in tumor cells has been viewed as critical to the development and sustainability of protective adaptive immune responses. To qualify as ICD, dying tumor cells must possess characteristics associated with immune signal 0 (danger) and signal 3 (inflammatory cytokines) that are required to instruct host DCs to take up tumor cell bodies, to mature and process these antigens into MHC-presented peptides, and to cross-prime antitumor T cells in a manner that results in the activation and expansion of cytotoxic T cells capable of emigration back to sites of disease.

In 2014, a group of key investigators from around the world working on ICD reached a consensus that there were at least three key feature molecules (DAMPs) required for the process of ICD. These include cell surface-exposed calreticulin, extracellular ATP and high mobility group box 1 (HMGB1), and/or the pathways allowing for their emission from dying cells, such as endoplasmic reticulum stress, autophagy, and necrotic plasma membrane permeabilization (35). When Zitvogel, Kroemer, and others originally proposed the concept, ICD included only the consideration of immunogenic apoptosis (36, 37). However, in 2013, our group, and that of Inoue and Tani, independently proposed that ICD includes not only immunogenic apoptosis but also necroptosis, autophagic cell death, and pyroptosis of cancer cells (30, 38). This extension has been validated by a number of recent studies. For example, necroptotic cancer cells induce ICD, and vaccination with such dying cancer cells induces efficient antitumor immunity (39). With a greater understanding of various mechanisms of cell death, the concept of ICD has continued to evolve. This year, Galluzzi and colleagues have further revised the ICD concept to now include additional types of cell death (such as necroptosis, pyroptosis) as we and other groups had originally proposed in 2013 (30, 38, 40).

A variety of therapeutic regimens and factors induce ICD in cancer cells (41). They include physical (radiotherapy and photodynamic therapy) (42), chemical (such as anthracyclines, oxaliplatin) (41), and biological ones. These biological agents include some OVs, immunogenic peptide (43), and other microorganisms and their products as they are potent PAMPs and more. We may arbitrarily think that infection with OVs automatically makes tumor cells highly immunogenic; however, this is not a guarantee as many viruses have evolved molecular mechanisms that subvert the exposure of DAMPs (such as ecto-CRT), thereby limiting the magnitude of ICD and thus consequent immune detection of such infected cells (12, 44). Indeed, such viruses induce cell death *via* non-immunogenic (sterile) apoptosis.

### OVs Induce *Bona Fide* ICD in Cancer Cells and May Interact Directly with Immune Cells, Leading to the Activation of Innate and Adaptive Immune Cells

Even though a variety of OVs have been shown to induce some features of ICD, few have been conclusively shown to represent *bona fide* inducers of tumor ICD. Based on the consensus-recognized ICD signature molecules (i.e., ecto-CRT, extracellular ATP, and HMGB1), only one OV thus far appears to meet the criteria for designation as an ICD-promoter: coxsackievirus B3 (45). However, a number of other OVs may also induce *bona fide* ICD, as they indeed serve to prime/induce adaptive antitumor immunity *in vivo*. The list is quite long and includes oncolytic adenovirus (46), influenza virus (47), HSV (25, 48, 49), measles virus (MeV) (50), NDV (51), VSV (5), and Sendai virus (52). However, we wish to emphasize that significantly more investigations will be required to validate such conjecture.

Some unarmed OVs possess the potential to activate innate and adaptive immunity. For example, an HSV-2 mutant, called ΔPK (due to the deletion of ICP10 that has protein kinase activity), has strong oncolytic activity for melanoma, induced mainly by a mechanism other than replication-induced cell lysis. It was found that it induced multiple non-redundant programmed cell death pathways (53). ΔPK inhibited the secretion of IL-10 from melanoma cells through virus replication and *c*-Jun *N*-terminal kinase/*c*-Jun activation. The virus-induced IL-10 inhibition led to enhanced cell surface expression of MHC class I chain-related protein A, the ligand for NKG2D receptor expressed on NK and CD8^+^ T-cells. Concomitantly, ΔPK also enhanced the secretion of TNF-α, GM-CSF, and IL-1β through autophagy-mediated activation pathways of Toll-like receptor 2 and pyroptosis and inhibited the expression of CTLA-4, one of the key negative immune checkpoint molecules (54).

Interestingly, ICD is not the only pathway by which OVs may modulate the host antitumor immune response. OVs may interact directly with immune cells to prime antitumor as well as antiviral immune responses. Reovirus may function as a PAMP interacting directly with DC, thus promoting DC maturation and stimulating the production of pro-inflammatory cytokines that may activate innate antitumor immunity (55). In contrast, reovirus may also infect tumor cells, leading to the (cross) priming of adaptive antitumor immunity (6). VSV can infect DC, leading to the improved capacity of these antigen-presenting cells to prime innate and adaptive antitumor immunity (56). The interaction of VACV with DC is a complex story. *In vivo*, both CD8^+^ and CD8^−^ DC are infected with VACV, resulting in the generalized upregulation in the expression of costimulatory molecules. However, IL-12 production is restricted to a subset of non-infected DCs (57). Interestingly, VACV may modulate the biological activity of another important immune cell type in the TME. Tumor-associated CD11b^+^Ly6G^+^ myeloid-derived suppressor cells (MDSCs) are normally immunosuppressive. Oncolytic VACV recruits MDSC with enhanced iNOS expression, which leads to beneficial antitumor activity. Depletion of iNOS-producing cells leads to very rapid tumor growth postvirus injection. These results suggest that the virus-induced iNOS^+^ MDSCs could represent an important antitumor effector cell population in the TME (58).

Many studies have shown that OVs elicited antitumor immunity, and this significantly contributed to the overall efficacy of the virus-mediated cancer therapy. As early as 1999, Toda et al. have shown that an oncolytic HSV (G207) could function as an *in situ* vaccine to induce specific antitumor immunity (25). An OV (e.g., MeV, Parvovirus H-1, or reovirus) would induce cancer cell oncolysis and allows DC to cross-prime tumor-specific CD8^+^ T cell response (6, 59, 60). As we will discuss later, arming the OVs with immune-stimulatory molecules would further promote eliciting potent antitumor immunity. A number of groups have shown that when the OVs encode a TAA, these OVs worked effectively as cancer vaccines (61–64). The antitumor immunity mounted by OVs have also been demonstrated in human cancer patients treated with oncolytic MeV (62, 65), HSV (66), AdV (67), and VACV(68).

### OVs Expressing Th1-Stimulatory Molecules

**Figure 1:**
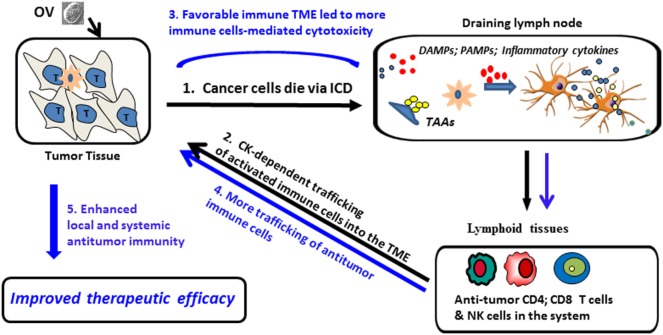
**Proposed model for ICD and pro-inflammatory cytokines/chemokines (Th1) promotion of oncolytic virus (OV)-mediated antitumor-immunity**. (1) OV infects tumor cells and induces ICD, leading to the release/presentation of signal 0 [damage-associated molecular patterns (DAMPs) and pathogen-associated molecular patterns (PAMPs)], along with tumor-associated antigens (TAAs) to dendritic cells (DCs), resulting in DC activation and Ag cross-presentation to antiviral and antitumor immune cells (activated NK cells, CD4^+^ and CD8^+^ T cells), followed by clonal expansion and maturation of antitumor T effector cells. (2) Cytokines/chemokines released during the acute inflammation in the tumor microenvironment (TME) promote trafficking of therapy-induced immune cells into the TME; (3) inflammation in the TME is sponsored by both viral- and tumor-reactive T cells, with immune-mediated eradication of tumor cells and tumor-associated stromal cells. Additional danger signals (signal 0), inflammatory cytokines, and chemokines (signal 3) and TAAs (signal 1) further activate tumor-associated DCs, overcoming local immunosuppression and prolonging the survival and functionality of antitumor immune cell populations; (4) reiterative rounds of DC-mediated cross-priming continue to allow for delivery of new (reinforcement) T immune effector cells into the TME (5) allowing for sustained antitumor efficacy within disseminated sites of disease.

To enhance the efficacy of antitumor immunity (Figure 1), many investigators have armed OVs with immune stimulatory genes. These may include costimulatory molecules, cytokines, and chemokines, such as IL-2, IL-12, IL-18, IFN-α/β, TNF-α, or GM-CSF, that are capable of promoting the development of cytotoxic immune effector cells.

All of these viruses are designed to further stimulate systemic antitumor immunity and to promote the trafficking of immune cells into the TME. Arguably, the best-studied OVs have been those armed with GM-CSF. The first such agent in the class approved by FDA is T-VEC, a HSV armed with GM-CSF for the treatment of patients with advanced-stage melanoma. Pexa-Vec, a VACV armed with GM-CSF, is currently being evaluated in a PHOCUS (phase III) global clinical trial in the setting of advanced-stage hepatocellular carcinoma (HCC). An additional oncolytic AdV armed with GM-CSF, designated CG0070, is being assessed for efficacy in a phase III clinical trial for the treatment of high-grade non-muscle invasive bladder cancer after failure to treatment with Bacillus Calmette–Guérin (BCG).

T-VEC represents a rationally designed OV to stimulate antitumor immunity based on engineering the viral vector to encode an immune-stimulatory gene. First, the virus was modified through deletion of two non-essential viral genes for replication in cancer cells (ICP34.5 and ICP47). ICP34.5 is a neurovirulence factor gene and its deletion attenuates viral pathogenicity and enhances tumor-selective replication (69). The second viral gene is ICP47, and its encoded protein enhances viral neurovirulence by limiting CD8^+^ T cell responses (70). Deletion of the ICP47 gene reduces viral-mediated suppression of antigen presentation and increases the expression of the HSV Us^11^ gene. The virus was then modified by insertion of cDNA encoding the cytokine GM-CSF. The infection of cancer cells by T-VEC induces ICD and local expression of GM-CSF, resulting in the recruitment, activation, and maturation of antigen-presenting cells, which are competent to promote tumor-specific T-cell responses (29).

Other OVs have been armed with chemokine genes. Expression of CCL5 (RANTES) from an OV has been shown to recruit DC, macrophages, NK, and CD8^+^ T cells into tumor sites, in association with the development of enhanced tumor antigen-specific CD8^+^ T cell and NK cell-mediated immune responses (71, 72). Recently, we have developed an OV encoding the chemokine CXCL11, designed to recruit CXCR3^+^ antitumor T effector cells and NK cells into the TME to mediate improve therapeutic efficacy (73). Although infection with this OV indeed led to these expected outcomes, we unexpectedly observed that vvDD-CXCL11 (but not parental vvDD) induced a systemic increase in tumor-specific IFN-γ-producing CD8^+^ T cells in treated animals. In an immunogenic tumor model, this therapy led to tumor regression and extended survival benefit, which was strictly dependent on CD8^+^ T cells and IFN-γ, but not CD4^+^ T cells (73). However, in a non-immunogenic tumor model, treatment with vvDD-CXCL11 monotherapy was not effective, necessitating its combination with a drug cocktail chosen for its ability to (re)condition the TME, which led to improved therapeutic efficacy in the MC38 colon tumor model (74).

Oncolytic virus expressing costimulatory molecules have also been explored. An oncolytic VACV expressing the 4-1BBL T-cell costimulatory molecule (rV-4-1BBL) was shown to be moderately effective in treating poorly immunogenic B16 melanomas in mice. Interestingly, when rV-4-1BBL treatment was combined with a lymphodepletion regimen, the authors observed enhanced tumor MHC class I expression, the promotion of viral persistence, and the rescue of effector-memory CD8^+^ T cells in association with improved therapeutic efficacy (75). When an oncolytic VACV was combined with an agonist antibody (Ab) specific for the costimulatory molecule 4-1BB (CD137), the dual treatment led to enhanced antitumor immunity and robust suppression of tumor growth in murine models (76). Enhanced immunity was associated with increased numbers of (CD11b^+^ and CD11c^+^) myeloid cells in tumor draining lymph nodes and enhanced infiltration of both NK cells and CD8^+^ T effector cells into the TME (76). Allison and associates have recently constructed an oncolytic NDV expressing the inducible costimulator and shown that when applied as an intratumoral therapy in combination with systemic CTLA-4 blockade, which treated mice exhibit enhanced infiltration of activated T cells in both virus-injected and uninjected, distal tumors that is curative in the B16-F10 tumor model (77).

We and other investigators in the field continue to search for new and exciting factors for inclusion in cutting-edge OV-based immunotherapies. In this regard, one of our groups has recently discovered the potent antitumor action of the IL-1 family member IL-36γ, which coordinately activates CD8^+^ T cells, NK cells, and Tγ/δ cells and synergizes with TCR activation and the type-1 polarizing cytokine IL-12 (78). When present within the TME, IL-36γ exerts profound antitumor activity *in vivo*, suggesting the great potential of this pro-inflammatory cytokine in OV-based cancer therapeutics.

### Combination of OV with Other Therapeutic Regimens/Drugs to Favorably Correct and Optimize the Immunologic TME

The cellular cross-talk between tumor cells and stromal cells within the TME, which is often mediated through soluble factors, creates an immunosuppressive environment that allows for enhanced viral replication and oncolytic activity in immune-deficient mice (79). The expression of VEGFR, which promotes tumor angiogenesis and progression, sensitizes the tumor vasculature to infection by oncolytic VACV (80). However, the TME coordinately inhibits protective antitumor immune responses that are crucial to the overall therapeutic efficacy of OVs applied to the immunocompetent (tumor-bearing) host. As a consequence, investigators have developed a variety of strategies including arming viruses with therapeutic genes or coapplying pharmaceutical interventions that promote ICD and/or that facilitate antigen cross-presentation in support of developing therapeutic antitumor T cell responses (81). We will discuss six strategies in this section.

#### Combination of OV with Conventional Chemotherapeutic Agents That Induce ICD

Many traditional chemotherapeutic agents possess the capacity to enhance host immunity (82). It is therefore logical to combine OV with this type of conventional drug to effect greater clinical benefit in the cancer setting. Combination treatments utilizing OVs and other pharmaceutical drugs have been reviewed extensively by Forbes et al. (83). We will discuss two recent studies to illustrate the most critical points. In the first study, the authors used autophagy stimulating or inhibitory drugs to determine if autophagy meaningfully impacts the outcome of oncolytic virotherapy. They showed that chloroquine or rapamycin significantly potentiate NDV-mediated oncolytic activity in mice bearing drug-resistant lung cancer (84). In this case, the exact mechanisms underlying treatment benefit remain to be elucidated. In another study, treatment with HSV-1 ICP0 null OV KM100 alone was determined insufficient to break immune tolerance in a breast tumor model; however, Workenhe et al. showed that by combining the virus with the ICD-inducing chemotherapy agent mitoxantrone, a significant survival benefit was gained for mice bearing Her2/neu TUBO-derived tumors. The take-home lesson was that such combination OV-based regimens coordinately enhances tumor immunogenicity, breaks immunologic tolerance established toward TAAs, and elicits superior therapeutic benefit (85).

#### Combination with Other Immunotherapies to Recruit and Sustain Protective Antitumor Immunity in the TME

By using tumor explant models, we investigated the impact of 3 in-clinic drugs for their ability to productively modulate the inflammatory characteristics of the TME: IFN-α, poly-I:C (a TLR3 ligand), and a COX-2 inhibitor (86–88). Tumor tissues reacted to individual drugs heterogeneously. A combination of IFN-α and poly-I:C uniformly enhanced the production of preferred (type-1 T cell recruiting) chemokines CXCL10 and CCL5, while reducing local production of CCL22, known to recruit suppressor cell populations. The addition of a COX inhibitor to this combination further enhanced these effects (86). We then applied this cocktail of agents to a colon tumor model in conjunction with the delivery of an oncolytic VACV. Sequential treatment with the virus vvCXCL11 and then the drug cocktail resulted in the upregulated expression of Th1-attracting CKs and a reduction in expression of the Treg-attracting CKs (CCL22 and CXCL12), in concert with enhanced trafficking of tumor-specific CD8^+^ T cells and NK cells into the TME. Notably, this combination regimen led to the greatest degree of therapeutic antitumor activity and to the long-term survival of the treated mice (74).

Another strategy is to engineer OV with a gene that serves as an antagonist to a dominant suppressor cell type or suppressor soluble mediator in the TME. MDSCs are one of the major regulatory cell subpopulations in the TME, where they promote tumor growth and progression (89). The inhibition of tumor-derived prostaglandin-E_2_ (PGE_2_) would be expected to block the induction of MDSCs and the recovery of NK cell activity (90). 15-Prostaglandin dehydrogenase (15-PGDH) is a tumor suppressor protein that is responsible for the degradation of PGE_2_. Walker et al. have constructed an oncolytic HSV expressing 15-PGDH and demonstrated that the delivery of this virus mitigates immune suppression and inhibits the growth of primary and metastatic breast cancer in a murine model (91). Recently, Hou et al. have also shown that an oncolytic VACV expressing this enzyme overcomes local immunosuppression, leading to profound changes in protective immune function within the TME. Such engineered OVs promote robust adaptive antitumor immunity and sensitize established and previously resistant tumors to regulation by immunotherapies (92).

#### Use a Vaccine Monotherapies or Combination Therapies

That OVs may function as effective cancer vaccines and impediments to their biologic activity have been discussed extensively in several recent reviews (30, 93–95). Here, we will focus on a discussion of prime-boost strategies as these relate to the use of OVs as cancer vaccines.

Heterologous prime-boost vaccination, a well-documented regimen to elicit robust CD8^+^ T cell responses, has been applied within the context of oncolytic immunotherapy. The first such study was carried out by Wan and colleagues, employing an antigen-expressing VSV and AdV. Intranasal delivery of the OV VSV-hDCT resulted in the activation of both CD4^+^ and CD8^+^ DCT-specific T-cells. These responses were significantly increased by subsequent booster vaccination using recombinant Ad (Ad)-hDCT. This regimen resulted in enhanced therapeutic efficacy against established B16-F10 melanomas in mice (96). In another study, the authors used recombinant VSV as a booster vaccine and demonstrated a massive increase in the secondary expansion of CD8^+^ antigen-specific T cells after priming with recombinant AdV (97). Vile et al. have also recently showed that a prime-boost vaccine regimen using distinct OVs (reovirus and VSV), when applied in combination with immune checkpoint blockade results in improved antitumor immunity/efficacy in the B16 melanoma model (98). Song, Kim, and others have developed a hybrid regimen using a complex of DNA and oncolytic AdV to treat malignant melanoma in a syngeneic mouse model (99). In this protocol, MART1 plasmid was used as a DNA-based vaccine to induce specific immunity, while the gene encoding murine GM-CSF and shRNA against mouse TGF-β2 were codelivered with MART-1 cDNA *via* an oncolytic AdV. This heterologous prime-boost vaccine strategy resulted in delayed tumor growth, likely resulting from (i) the induction of anti-MART1 T effector cells, (ii) enhanced antigen-presentation driven by GM-CSF and TGF-β2 shRNA, (iii) tumor growth inhibition by TGF-β2 shRNA, and (iv) tumor cell-specific OV-induced oncolysis (99).

#### Combination with CAR T Cell-Based Adoptive Immunotherapy

CAR T cells represent one of the most promising new approaches in cancer immunotherapy (100), with only a single study thus far integrating OV (101). In this report, an oncolytic Ad (Ad5Δ24) was armed with chemokine genes CCL5 and IL-15 and applied as a recruiter (*via* CCL5) and sustainer (*via* IL-15) of CAR-T cells (reactive against the tumor-associated ganglioside GD2) into/within the TME. Application of the OV was observed to enhance the function of CAR T cells *in vivo*, with the combination immunotherapy extending overall survival in mice bearing neuroblastomas.

#### Combination with Bispecific T-Cell Engagers (BiTEs)

So far two studies have explored this novel approach. Song and associates constructed an oncolytic VACV encoding a secretory BiTE composed of two single-chain variable fragments specific for CD3 and the tumor cell surface antigen EphA2 (EphA2-TEA-VV) (102). This virus, when combined with human T cells, exhibited potent antitumor activity in a lung cancer xenograft model. Earlier this year, Alemany and associates generated an oncolytic AdV encoding a BiTE (cBiTE) coordinately targeting EGFR and CD3 (ICOVIR-15K-cBiTE). Intratumoral injection of this recombinant AdV increased the persistence and accumulation of tumor-infiltrating T cells *in vivo*. This OV, when combined with peripheral blood mononuclear cells or T cells exhibited enhanced antitumor efficacy (103). The results from these two studies suggest that BiTE-armed OVs may overcome some key limitations associated with current oncolytic virotherapy-based strategies.

#### Combination with Complement Inhibition

Natural barriers in the blood, including neutralizing antibodies and complement, likely limit our ability to repeatedly administer the same OVs intravenously. As a consequence, it makes sense to consider means by which to coordinately inhibit complement activation to improve the utility and antitumor efficacy of OV-based immunotherapies. We showed that inhibitors of C5 complement enhanced the infection of cancer cells by VACV *in vitro*, even in the absence of antivaccinia antibodies (104). In a recent study, Evgin et al. demonstrated that in immunized rats, complement depletion stabilized VACV in the blood, resulting in the improved delivery of virus into the TME (105).

#### Combination with Immune Checkpoint Blockade

Immune checkpoint blockade-based immunotherapy has made major advances over the past several years, to now become standard of care in the setting of many forms of cancer. Since the anti-CTLA4 Ab (ipilimumab) was FDA approved for use in patients with advanced-stage melanoma in 2011, immune checkpoint antagonists (including anti-CTLA4 and anti-PD-1/PD-L1 antibodies) have now been approved for use against six forms of cancer. Immune checkpoint molecules are a natural means used by the immune system to maintain homeostasis, ensuring self-tolerance and the prevention of pathologic autoimmunity. In tumor tissues, however, these signals are often upregulated, allowing for progressively growing tumors to evade local protective immune responses (106).

Despite enthusiasm for the continued clinical use of immune checkpoint blockade as a general strategy to combat cancer, this approach works best in patients who exhibit existing evidence of ongoing antitumor immune responses, and it fails in cases where the TME is devoid of a protective immune signature. Furthermore, even in the setting of advanced-stage melanoma, only 15–25% patients exhibit durable objective clinical responses. Thus, there exists obvious potential for synergy between therapeutic regimens using OVs and immune checkpoint blockade. Mechanistically, OVs offer the possibility of priming, boosting, and recruiting effector T cells into the TME, where immune checkpoint blockade may serve to enhance/sustain the potency of antitumor TIL *via* the removal of inhibitory signals (94, 107).

In such combination immunotherapies, the immune checkpoint antagonist Ab could be physically delivered as a protein or encoded by a recombinant OV used to infected cancer cells. The first study for such a combination approach was published by Hemminki and his team in 2012, demonstrating that targeted cancer immunotherapy could be achieved using an oncolytic AdV encoding a fully humanized monoclonal Ab reactive against CTLA-4 (108). Since then, several original research papers on this exciting combination strategy have been published (109–118).

Zamarin and others demonstrated in mouse models that localized immunotherapy with oncolytic NDV combined with anti-CTLA4 Ab could cure the majority of treated tumor-bearing mice, while treatment with NDV alone was effective in only 10% of cases. Importantly, this combinatorial strategy was observed to induce an immune response against both virally infected and control, uninfected tumors, with minimal reactivity noted against unrelated, third-party tumors. Interestingly, the antitumor efficacy of this approach was dependent on CD8^+^ T-cells, NK cells, and type I IFN, but not on oncolysis. Treatment with this combination of oncolytic NDV and anti-CTLA4 Ab led to systemic tumor rejection and subsequent protection of the host against tumor rechallenge in poorly immunogenic tumor models (111).

Vile and colleagues have used a prime-boost vaccine regimen with separate OVs in concert with immune checkpoint blockade to further improve antitumor efficacy in combination approaches (98). They hypothesized that reovirus-induced CD8^+^ antitumor T cell responses, when combined with the VSV-ASMEL-induced CD4^+^ Th17 responses, would result in potent antitumor immunity/efficacy. In their study, tumor-bearing mice were first treated with intratumoral injection of reovirus, followed by intravenous delivery of VSV-ASMEL. This regimen significantly improved the overall survival of mice bearing subcutaneous B16 melanoma. Finally, the triple combination immunotherapy significantly enhanced survival of mice, with improved frequencies of durable cures (versus mono- or dual-component treatment cohorts), in association with robust Th1 and Th17 immune responses against tumor antigens (98).

In our recent study, we explored the efficacy of combined therapy using oncolytic VACV and anti-PD-L1 Ab in murine colon and ovarian cancer models (118). We hypothesized that an oncolytic VACV would elicit antitumor adaptive immune response and attract T cells into the tumor, with the resulting inflammation promoting PD-L1 expression in both cancer and immune cells, making the TME susceptible to subsequent treatment with the anti-PD-L1 antagonist Ab. We determined that the combination immunotherapy facilitated tumor infiltration of effector CD8^+^ and CD4^+^ T cells (expressing IFN-γ, ICOS, granzyme B, and perforin), while reducing the prevalence of PD-L1^+^ cells and exhausted PD-1^+^CD8^+^ TIL in the TME. The combination protocol also resulted in superior antitumor efficacy (versus the component monotherapies) and extended overall survival. We predict that these combination OV/immune checkpoint blockade-based immunotherapies will expand the use of checkpoint inhibition to a much wider population of cancer patients (118).

## Clinical Studies with OVs

Starting in the year 2000, a variety of OVs have been tested in clinical trials. Many phase I studies with a variety of OV have been conducted, mostly dealing with safety and feasibility issues. Some OVs have been tested in phase II or beyond. Since 2010, nine phase II/III clinical trials employing four types of OVs have been reported (Table 1). In this section, we will focus on those OVs in phase II trials and then briefly discuss the OVs with completed phase III trials, and two phase Ib clinical studies combining T-VEC with immune checkpoint blockade in patients with advanced melanoma.

**Table 1:** **Phase II clinical trials in cancer patients with oncolytic viruses (OVs) (from year 2010 to current)**.

OV	Combination or others	Cancer type (patient number)	Primary endpoints	Clinical responses	Reference
Herpes simplex virus (HSV) (NV1020)		Refractory metastatic colorectal cancer (19 in phase II)	Toxicity and efficacy	50% patients with stable disease	Geevarghese et al. (119)

HSV (T-VEC)		Metastatic melanoma patients (50)	Local and distant antitumor immunity	(1) Elevated levels of regulatory T cells (Tregs), suppressor T cells (Ts), and myeloid-derived suppressor cell (MDSC) within established tumors (2) Direct injection of T-VEC induces local and systemic antigen-specific T cell responses and decreases Treg, Ts, and MDSC in patients exhibiting therapeutic responses	Kaufman et al. (66)

HSV (T-VEC)	Radiotherapy + cisplatin	Untreated stage III/IV squamous cell carcinoma of the head and neck (17)	Safety and efficacy	(1) 82% patients showed tumor responses by RECIST (2) 93% pathologic complete regression (3) DFS 82% at 29 months	Harrigton et al. (120)

HSV (T-VEC)	Systemic versus local responses	Stage IIIc or IV melanoma (50)	Comparison of efficacy in directly injected lesions, and uninjected non-visceral and visceral lesions	(1) Lesions directly injected: 67% decreased in size; 46% completely resolved (2) Uninjected non-visceral lesions: 41% decreased in size; 30% completely resolved	Kaufman et al. (121)

Reovirus (RT3D; same as Reolysin^®^)	Carboplatin + paclitaxel	Advanced malignancies (31)	Safety and efficacy	(1) No dose-limiting toxicity (2) One complete response, 6 partial responses, 9 stable disease, and 8 disease progression	Karapanagiotou et al. (122)

Reovirus (Reolysin^®^)		Advanced melanoma (21)	Safety and efficacy	(1) Viral replication (2/21) (2) No objective response (3) Median time to progression and survival were 45 and 165 days	Galanis et al. (123)

Reovirus (Reolysin; Pelareorep)	Paclitaxel/carboplatin	Metastatic pancreatic adenocarcinoma (arm A, *n* = 36)		(1) The majority of PFS time was without toxicity or progression (4.3 months) (2) Patient immunophenotype appeared important (3) Overall, pelareorep was safe but does not improve PFS	Noonan et al. (124)

AdV	Radiation	Intermediate-risk prostate cancer -(21 in the arm)	Acute (≤90 days) toxicity	When used combined, a clinically meaningful reduction in positive biopsy results at 2 years	Freytag et al. (125)

Vaccinia virus (JX-594; Pexa-Vec)		Advanced hepatocellular carcinoma (*n* = 30)	To determine the optimal dose	(1) JX-594 replication and granulocyte-macrophage colony-stimulating factor expression preceded the induction of anticancer immunity (2) Median survival of 14.1 months compared to 6.7 months on the high and low dose, respectively	Heo et al. (68)

Two oncolytic HSVs have now been tested in four phase II trials treating patients with three different types of cancer. In the first trial, NV1020 was evaluated in patients with pretreated refractory metastatic colorectal cancer, where treatment was observed to stabilize liver metastases with minimal toxicity (119). In a second trial, Kaufman et al. assessed local and systemic immune responses after T-VEC was injected directly into melanoma lesions. They determined that (i) established tumors contained elevated levels of Treg, suppressor T cells (Ts), and MDSC at baseline and (ii) T-VEC treatment enhanced local and systemic antigen-specific T cell responses in association with decreased levels of Treg, Ts, and MDSC in those patients who exhibited objective clinical responses to therapy (66). In a third study, T-VEC was combined with radiotherapy and cisplatin for the treatment of patients with untreated stage III/IV squamous cell carcinoma of the head and neck (SCCHN) (120). Finally, Kaufman and others compared the efficacy of intratumorally delivered T-VEC versus non-injected non-visceral or visceral lesions. They found that the therapeutic efficacy was greatest in the injected lesions, intermediate in non-injected non-visceral lesions, and lowest in visceral lesions (121).

Reovirus has also been evaluated in three phase II trials treating various advanced forms of cancer. Reolysin (RT3D) administered with carboplatin and paclitaxel has been evaluated for safety and efficacy in patients with SCCHN (122). The authors report no dose-limiting toxicity, with a large fraction of patients exhibiting stable disease, as well as, several PR or CR (4%). Reolysin has also been used to treat patients with advanced-stage melanoma *via* intravenous delivery, where again the treatment was observed to be well tolerated, with evidence for virus replication in tumor biopsies (123). Furthermore, Reolysin has been applied alone or in combination with paclitaxel/carboplatin for the treatment of patients with metastatic pancreatic adenocarcinoma in a randomized phase II trial (124). The approach was found to be safe, although the combination therapy was not superior to carboplatin/paclitaxel alone in improving patient progression free survival.

Other OVs have also been tested in phase II clinical trials. Oncolytic AdV applied in combination with radiation has been used to treat intermediate-risk prostate cancer in a prospective randomized phase II trial (125), clinically meaningful reductions in positive biopsies noted at 2 years posttreatment. To date, the most encouraging results have been obtained in a trial using Pexa-Vec (JX-954) to treat patients with liver cancer (68), where coordinate viral replication and GM-CSF expression in tumors was observed, with therapy-induced antitumor immunity also being detected. In this trial, the duration of patient survival was directly related to viral dose, with a median survival of 14.1 months in the high-dose cohort versus 6.7 months in the low-dose group.

Four phase III trials involving administration of OVs have been completed or remain open to patient accrual at this time. H101 is a recombinant human AdV type 5 with E1B deletion (presumably) conferring conditional replication in p53-deficient cancer cells. China approved the clinical use of H101 in 2005 (Oncorine^®^). In a multicenter, open, randomized, and parallel controlled clinical study, H101 combined with chemotherapy was reported to be superior to chemotherapy alone with a good safety profile in patients with squamous cell carcinoma of head and neck and the esophagus (126). In the United States, T-VEC was used in a successfully completed phase III OPTiM study and was FDA approved for the treatment of patients with advanced-stage melanoma in 2015 (127). A third OV, Pexa-Vec (JX-594), is currently being investigated in a worldwide phase III PHOCUS trial in patients with hepatocellular carcinoma. Finally, CG0070 (AdV expressing GM-CSF) is currently being evaluated in BOND2, a phase III pivotal study, examining treatment efficacy against high-grade, non-muscle invasive bladder cancer after failure to BCG therapy.

As we have discussed previously, positive clinical results were obtained from HCC patients in South Korea receiving Pexa-Vec (68). In contrast, its TRAVERSE Phase 2 study of Pexa-Vec in second-line advanced liver cancer in the United States (in 2013) did not meet its primary endpoint. It will be interesting to analyze the contradictory results in Asia and North America in greater detail. We would argue that the success of OV, as a form of immunotherapy, critically depends on intrinsic or therapeutically inducible cancer immunogenicity. In Asia, infection with hepatitis B virus is the more common cause of HCC, while in the United States, hepatitis C virus (HCV) is a more common etiologic agent. HCV may also possess a higher capacity to evade the immune system (128). As of today, we still do not have an effective vaccine against HCV, and we would hypothesize that liver cancers (mostly HCC) resulting from chronic HCV infection may be generally less immunogenic than those tumors caused by HBV infection.

We have been developing the WR strain VACV as an OV (129). Phase I clinical trials with vvDD, a double viral genes-deleted tumor-selective OV, have now been completed. The first-in-human dose-escalation trial of vvDD was performed in 16 patients with advanced-stage solid tumors, predominantly colorectal cancer (130). Viral dose escalation, delivered intratumorally, proceeded without dose-limiting toxicities, up to a maximum feasible dose at 3.0e9 pfu. Viral replication in tumors was reproducibly observed, with virus recovered from both injected and non-injected tumors. In summary, vvDD delivered intratumorally was well tolerated in patients, with viral administration leading to selective infection of injected and non-injected tumors, with coordinate antitumor activity noted. In a second trial, we delivered the virus intravenously into cancer patients (131). Again, we observed no dose-limiting toxicities or treatment-related severe adverse events. Viral genome DNA was detectable in patient blood shortly after virus administration, with prolonged viral replication detected in tumor tissues isolated from two patients. Viral replication was not found in non-tumor tissues, with the exception of sites of injury. It is worth noting that the best clinical responses were observed in the two patients with melanoma in these two trials. This could reflect the consensus that melanoma is a particularly immunogenic type of cancer (and possibly preferred target for immunotherapy (132)) and/or that skin is the normal target tissue for infection by VACV (possibly making it easier for VACV to induce ICD in cutaneous forms of cancer).

Szalay, Fong, and others have also been developing LIVP strain-derived oncolytic VACV GLV-1h68 (commercial name: GL-ONC1) (133). Multisite clinical studies have demonstrated a favorable safety profile and hinted at the potential use of GL-ONC1 as an effective therapeutic agent (e.g., ASCO Annual Meeting 2015). Two ongoing phase I clinical trials are currently evaluating i.v.-administered GL-ONC1 along with concurrent chemoradiotherapy for patients with locoregionally advanced head and neck carcinoma and intrapleural administration of GL-ONC1 for patients with malignant pleural effusion.

At this time, the most exciting clinical studies appear to be those combining OV with immune checkpoint blockade. A phase Ib study using T-VEC with ipilimumab, an anti-CTLA-4 antagonist Ab, in patients with unresectable stage IIIb/IV melanoma has been recently reported (134). Nineteen patients were included in the safety analysis. No dose-limiting toxicities were observed. The objective response rate reached 50%, with 44% of patients exhibiting durable responses lasting ≥6 months. The conclusion of the study was that the combined treatment had a tolerable safety profile and appeared to have greater efficacy than either monotherapy.

Pembrolizumab is an anti-PD-1 antagonist Ab. Previous clinical studies have shown that clinical administration of this Ab leads to greater progression-free survival and overall survival than ipilimumab in melanoma patients, suggesting that a combination of T-VEC with pembrolizumab might be more effective than the combination with ipilimumab. An ongoing phase I–III study was designed to explore this combination for patients with unresected melanoma (NCT02263508) (135). In the phase Ib study of 21 patients, the reported ORR was 57%, with 24% of patients with confirmed complete response. The disease control rate was 71%. A phase III randomized, double-blind, placebo-controlled trial (MASTERKEY-265) is now planned for 660 patients with unresectable state IIIb/IV melanoma.

## Conclusion and Perspectives

The TME in the setting of advanced-stage cancers is highly immunosuppressive (136). As we and others have previously suggested, this immunosuppressive property poses a double-edge sword in consideration of OV-based immunotherapy. Such suppression limits immune regulation of viral replication in support of direct oncolysis, but it represents a major impediment to the development, targeting and operational integrity of protective antitumor immunity that appears crucial to the durable clinical success of OV-based interventional strategies. How we manipulate this delicate balance may likely determine the optimal benefits that can be achieved using such treatment modalities in the clinic. Notably, administration of OVs often leads to ICD of cancer cells, a process in which dying tumor cells expose/release multiple potent danger signals (signal 0) and pro-inflammatory cytokines (signal 3), while in some cases, coordinately upregulating their expression of MHC class I and II antigens. ICD in the TME begets efficient tumor antigen-cross presentation (signals 1 and 2) by tumor-associated DC that serve as the instigators of robust type-1 T cell responses capable of limiting tumor growth/metastasis. Combinatorial OV-based approaches allow for the fine tuning of the immune microenvironment within tumors, leading to removal of suppressive cells/factors and the recruitment and maintenance of therapeutically induced antitumor immune cells. Such combinational approaches, incorporating chemotherapeutic drugs, vaccines, or adoptive immune cell therapies, hold great clinical promise in optimizing the therapeutic potential of OV-based interventional approaches.

There also remains great need to further investigate mechanisms underlying patient resistance to oncolytic immunotherapy and any OV-associated toxicities. There are primary, adaptive, and acquired resistance to OV-mediated and other cancer immunotherapy (137). As our understanding for mechanisms of resistance continues to improve, we will be in position to rationally design combinatorial strategies to safely overcome such resistance. Our recent study combining OV and anti-PD-L1 represents one such study (118). There is also need to define biomarkers associated with clinical response (or resistance, toxicity) to treatment with oncolytic immunotherapy. Only a few studies have been published in this area of research to date. In this regard, serum HMGB1 has been shown to be a predictive and prognostic marker for successful oncolytic immunotherapy with AdV (138). In another study, immunoglobulin-like transcript 2 has been identified as a biomarker of therapeutic response to oncolytic VACV (13). These types of studies may enable us to better predict OV-based treatment outcomes in future clinical trials.

A number of hurdles remain that limit wide-spread use of OV-based therapies in the cancer setting. The first hurdle is the inability of OV to efficiently deliver and propagate throughout the entire tumor and to infect cancer cells that are at extended distances from the site of virus injection or from the original site of infection site after systemic delivery, which limits the ability of this approach to achieve consistent therapeutic responses in patients with disseminated disease. The tumor matrix also hinders virus diffusion throughout a given lesion. Some suggested means to circumvent this limitation have been offered. For example, the engineered overexpression of matrix metalloproteinases-1 and -8 significantly depletes tumor-associated sulfated glycosaminoglycans, resulting in increased tumor perfusion and greater distribution of injected virus in association with improved therapeutic efficacy (139). Similarly, enforced expression of hyaluronidase by OV led to improved virus spread throughout the tumor and to greater therapeutic benefit (140). Losartan, an angiotensin II receptor antagonist, appears capable of enhancing the distribution and efficacy of nanomedicines, including OVs (141). Another reason for the low efficiency of virus distribution throughout the tumor reflects the relatively high interstitial fluid pressure of the TME (142). In this regard, blood flow may affect the intratumoral extravasation of systemically delivered OVs. Indeed, one recent study demonstrated that perfusion pressure greatly affects the intratumoral extravasation of OVs (143). Antiangiogenic therapies, through their induction of collagen degradation, can also enhance intratumoral distribution of oncolytic AdV (144). Clearly, additional investigations will be required to further improve upon tumor uptake and intralesional distribution of OVs to yield more effective cancer therapies.

A second hurdle involves the need to develop a broad repertoire of therapeutic immune cells that circulate systemically to impact disseminated disease, which typically evolves over time (145). Such timing can be adversely affected by antiviral immunity that may clear the OV prematurely, thus reducing therapeutic efficacy. For example, HSV-mediated oncolytic virotherapy for glioblastoma is often improved with the suppression of innate immune responses, leading to increased viral replication and subsequent oncolysis (146, 147). However, the boosting of antiviral immunity has also been shown to be required for efficient OV-mediated therapy benefits in some tumor models (7, 148, 149) and can play a “helper” role in the evolution of adaptive antitumor immunity elicited by OV, with the ultimate therapeutic efficacy requiring a delicate balance of the avidity, potency, and timing of the immune response directed against the virus versus the tumor (150).

A third hurdle reflects toxicities associated with OVs. In patients receiving Imlygic (T-VEC), adverse reactions, including fatigue (50%), chills, pyrexia, nausea, influenza-like illness, injection-site pain, and vomiting, occurred in over 20% of treated patients, with the most common grade 3/4 adverse reaction being cellulitis (127). Given the use of a live virus, Imlygic can cause life-threatening dissemination of herpetic infections in immunocompromised patients. As a result, the use of T-VEC is contraindicated in immunocompromised patients and in pregnant women. We have recently evaluated OV derived from the WR strain of VACV (vvDD), and based on our findings, patients with actively healing wounds, or those with acute inflammatory conditions involving the skin or oral mucosa, should be excluded from using this OV (131). It also should not be used by immunocompromised patients.

Finally, accumulating evidence suggests that microbiota play an important role in the initiation, progression, and dissemination of cancer, not only at epithelial barriers but also in sterile tissues. Perhaps more importantly, barrier tissue microbiota can modulate cancer patient response to interventional therapy (including immunotherapy), as well as, patient adverse events to therapy (151). In this regard, it will be critical to further study the relationship between OV and microbiota in the host to better predict the likelihood of therapeutic efficacy versus treatment-associated toxicity.

In summary, it is indeed an exciting time to work in field of cancer immunotherapy. By combining with other forms of cancer immunotherapy, especially modulation of immune checkpoint pathways (impacting signal 2) and adoptive cell therapies, the future appears bright for the effective use of OV-based immunotherapy in the cancer setting.

## Author Contributions

ZG collected and read relevant papers and designed and drafted the manuscript. WS revised and polished the whole manuscript. All other authors have made suggestions to the manuscript. All authors have read and approved the final manuscript.

## Conflict of Interest Statement

The authors declare that the research was conducted in the absence of any commercial or financial relationships that could be construed as a potential conflict of interest.
